# Mitophagy in Antiviral Immunity

**DOI:** 10.3389/fcell.2021.723108

**Published:** 2021-09-03

**Authors:** Hongna Wang, Yongfeng Zheng, Jieru Huang, Jin Li

**Affiliations:** ^1^Affiliated Cancer Hospital and Institute of Guangzhou Medical University, Guangzhou, China; ^2^Key Laboratory of Cell Homeostasis and Cancer Research of Guangdong Higher Education Institutes, Guangzhou, China; ^3^GMU-GIBH Joint School of Life Sciences, Guangzhou Medical University, Guangzhou, China

**Keywords:** mitophagy, virus, mitochondria, infection, autophagy, immune

## Abstract

Mitochondria are important organelles whose primary function is energy production; in addition, they serve as signaling platforms for apoptosis and antiviral immunity. The central role of mitochondria in oxidative phosphorylation and apoptosis requires their quality to be tightly regulated. Mitophagy is the main cellular process responsible for mitochondrial quality control. It selectively sends damaged or excess mitochondria to the lysosomes for degradation and plays a critical role in maintaining cellular homeostasis. However, increasing evidence shows that viruses utilize mitophagy to promote their survival. Viruses use various strategies to manipulate mitophagy to eliminate critical, mitochondria-localized immune molecules in order to escape host immune attacks. In this article, we will review the scientific advances in mitophagy in viral infections and summarize how the host immune system responds to viral infection and how viruses manipulate host mitophagy to evade the host immune system.

## Introduction

Mitochondria, the energy-generating organelles of eukaryotic cells, are encased in a unique double-layered membrane in which energy from organic substances is converted into ATP by oxidative phosphorylation to support cell survival (Friedman and Nunnari, 2014). In addition to its role in metabolism, the mitochondrion also acts as a signaling platform for apoptosis and innate antiviral immunity (Weinberg et al., 2015; Banoth and Cassel, 2018). The process of mitochondrial oxidative phosphorylation generates large amounts of by-products called reactive oxygen species (ROS), which can lead to mitochondrial damage. If not repaired or removed in a timely manner, large numbers of damaged mitochondria will accumulate in the cell, increase cell burden, and cause loss of cellular function. In addition, the crucial role of mitochondria in apoptosis also requires that the quality of these organelles be strictly controlled to avoid cell death (Matthew et al., 2021). A cellular strategy known as mitophagy functions to remove damaged mitochondria. Mitophagy is an evolutionarily conserved quality-control mechanism that selectively targets damaged or excess mitochondria to the lysosomes for degradation and plays an important role in maintaining the number of mitochondria (Youle and Narendra, 2011). In specific cells (e.g., mature red blood cells), selective elimination of mitochondria is essential for cellular function. Therefore, mitophagy is necessary for the degradation of excess mitochondria to maintain cellular homeostasis and survival (Montava-Garriga and Ganley, 2020).

In recent years, increasing evidence shows that viruses promote their own replication and propagation in host cells by hijacking mitophagy (Xia et al., 2014b; Zhang et al., 2018; Cho et al., 2020). Some viruses can trigger mitophagy directly or indirectly and control the mitophagic process through various strategies (Lazarou, 2015; Xu et al., 2020). Virus-induced mitophagy results in degradation of the antiviral signaling protein MAVS localized in the outer mitochondrial membrane (OMM), thereby suppressing host immune signaling (Gkikas et al., 2018). In recent years, tremendous efforts have been made to advance the understanding of the relationship between virus-induced mitophagy and host immunity. This review will summarize these recent advances and provide a parsing of the mechanisms of mitophagy and the role of mitophagy in antiviral immunity. We hope the insights into the molecular mechanisms of viral-induced mitophagy summarized in this review are valuable for the development of novel antiviral strategies.

## An Overview of Mitochondria

### The Structure and Function of Mitochondria

Mitochondria are one of the most important organelles in eukaryotic cells, and they are widely believed to be bacterial in origin (Gray, 2012). This theory, which is known as the endosymbiotic hypothesis, suggests that mitochondria are descendants of bacteria that were engulfed by another prokaryote or another type of cell during evolution. The mitochondria that thus evolved formed a structure enclosed by an inner and an outer membrane and contained four functional regions: the outer membrane, inner membrane, intermembrane space, and matrix, as well as genetic material independent of the nucleus (Friedman and Nunnari, 2014). The outer membrane of the mitochondria is flat and serves as both a limiting membrane and a platform for material exchange. The inner membrane folds and extends from the outside to the inside, forming a series of structures called cristae. Cristae increase the surface area of the inner membrane, and their dynamics allow the mitochondria to adapt to different states of the cell (Cogliati et al., 2016; Pernas and Scorrano, 2016). This structure supports the oxidative phosphorylation function of mitochondria, making them an important component of metabolism and signal transduction.

In addition to their functions in metabolism, mitochondria are central organelles of the apoptosis pathway. Upon initiation of apoptosis, cytoplasmic Bax translocates to the mitochondria and cooperates with mitochondria-localized Bak to trigger the release of cytochrome c from the mitochondria to the cytoplasm, which activates caspases to execute apoptosis (Green and Levine, 2014; Pickles et al., 2018). In addition, MAVS on the OMM plays an important role in regulating viral-triggered host innate immunity. Upon viral invasion, the cellular pattern recognition molecules RIG-I and MDA5 sense viral RNA and oligomerize and activate the MAVS, which leads to the production of antiviral cytokines by the cell and ultimately drives viral clearance by the immune response (Tiku et al., 2020; Rai et al., 2021).

### Mitochondria and Antiviral Immunity

Pathogen-related molecular patterns (PAMPs) are conserved structures that are common to pathogenic microorganisms but not present in the host. Damage-related molecular patterns (DAMPs) are endogenous substances released from damaged or dying cells into the intercellular space or blood circulation. The natural immune system employs a limited number of pattern recognition receptors (PRRs) to recognize PAMPs and DAMPs and activate intracellular signal transduction and gene expression, resulting in the release of cytokines and chemokines that trigger the immune response (Sumpter and Levine, 2010; Banoth and Cassel, 2018).

During viral invasion, mitochondria are involved in host immunity in two ways. First, they provide essential intermediates for the activation of the antiviral inflammasome. Following viral infection, the damaged mitochondria release substances such as mitochondrial DNA (mtDNA) and mitochondrial ROS (mROS) into the intracellular spaces (Li et al., 2007, 2018; Cheng et al., 2014; Wang R. et al., 2019; Ledur et al., 2020). Free mtDNA or mROS in turn activate the NLRP3 inflammasomes, leading to the secretion of caspase-1-dependent pro-inflammatory cytokines IL-1β and IL-18. The mtDNA also activates the cGAS-STING pathway to promote type I interferon (IFN) production via activation of the TBK1-IRF3 signal pathway (Khan et al., 2015; Banoth and Cassel, 2018). Second, the mitochondrial outer membrane also hosts MAVS, an essential component of the innate immune pathway against RNA viruses (Banoth and Cassel, 2018). Upon exposure to RNA viruses, RIG-I located on the host cell surface undergoes a conformational change, exposing the N-terminal caspase recruitment domain (CARD), which interacts with the CARD domain of MAVS, leading to MAVS activation. Activated MAVS drives IFN expression through recruitment and activation of TRAF6, TANK-binding kinase 1 (TBK1), and several other molecules. In turn, MAVS also activates the NLRP3 inflammasome, which promotes the secretion of IL-1β and IL-18, triggering an inflammatory response (Castanier et al., 2010).

### Maintenance of Mitochondrial Homeostasis

Under physiological conditions, mitochondria are dynamically interconnected, undergoing constant fusion and fission. The balancing act between fusion and fission is critical to maintaining mitochondrial homeostasis and function. Fusion is driven by three GTPases, namely, mitofusin 1 (MFN1), mitofusin 2 (MFN2), and optic atrophy 1 (OPA1), where MFN1 and MFN2 are responsible for fusion between OMMs and OPA1 is responsible for fusion between inner mitochondrial membrane (IMM) (Matthew et al., 2021). MFN1 and MFN2 are large GTPases that traverse the OMM twice. When bound to other copies of themselves on two adjacent mitochondria, MFNs bring the adjacent mitochondria together. Once MFNs are tethered, they hydrolyze GTP to catalyze the remaining OMM fusion process. OPA1 is responsible for mitochondrial inner membrane fusion. Under physiological conditions, OPA1 is hydrolyzed by a protease to form a long L-OPA1 anchored to the IMM and a soluble short S-OPA1. L-OPA1 and S-OPA1 are required to act synergically to ensure proper fusion of the IMM. Mitochondrial fission is coordinated by another GTPase DRP1 (also known as DNM1L), which is recruited from the cytoplasm to the OMM when activated. Active DRP1 assembles on the outer membrane into an oligomeric structure that wraps around the fission site and then hydrolyzes GTP to drive the shrinkage and rupture of the mitochondrial membrane for final fission (Cogliati et al., 2016; Fenton et al., 2021).

Fusion helps connect individual mitochondria to form an extensive, interconnected network, facilitating the exchange of materials between the mitochondria and restoring function to partially damaged mitochondria (Pernas and Scorrano, 2016). When mitochondria are damaged beyond repair, they must be eliminated by mitophagy. However, given that the tubular mitochondria are beyond the wrapping capacity of autophagosomes (which are only 500–1,500 nm in diameter) for enclosure, they must split into small granular mitochondria (with an average size of 500–1,000 nm) in order to be engulfed and degraded by autophagosomes (Mizushima et al., 2002). Therefore, mitochondrial fission is considered a prerequisite step for mitophagy (Mizushima et al., 2002; Rambold et al., 2011; Pernas and Scorrano, 2016; Burman et al., 2017). In response to the apical role of the mitochondria in antiviral immunity and the critical role of fusion and fission in maintaining mitochondrial health and function, viruses have developed a range of strategies to influence mitochondrial dynamics and promote their own proliferation and persistence in the cell (Ren et al., 2020).

## Overview of Autophagy

Autophagy is a natural, conserved process by which excess or dysfunctional cellular components are degraded through lysosomes. Although initially characterized as a degradation pathway induced to protect cells from starvation, it has become clear that autophagy plays a major role in maintaining homeostasis in non-starved cells. Three types of autophagy have been identified so far: macroautophagy, microautophagy, and molecular chaperone-mediated autophagy. Among them, macroautophagy is the most well characterized; therefore, it is usually referred to simply as autophagy (Mizushima and Komatsu, 2011). Therefore, in the following, we refer to macroautophagy when we describe autophagy.

Autophagy consists of the sequential formation of membrane vesicles, including the phagophore, autophagosome, and autolysosome. After the induction of autophagy, free membrane structures appear in the cell cytoplasm and form phagophore, a cup-shaped double-membrane vesicle. The phagophore gradually expands and becomes a closed double-layer membrane structure enclosing damaged organelles and part of the cytoplasm. This double-layered membrane structure is called autophagosome. The outer membrane of the autophagosome fuses with the lysosome membrane to form an autolysosome. Under the action of the acidic environment and lysosomal enzymes, the inner membrane and its encapsulated materials are degraded. Finally, the products of degradation, such as amino acids, nucleotides, and free fatty acids, are reused to synthesize macromolecular or generate energy (Klionsky et al., 2016).

With the rapid development in genetic technologies, the molecular mechanisms involved in autophagy were first identified in yeast, and subsequently, similar mechanisms were identified in mammals. To date, nearly half of the autophagy-related genes (Atg) have been found to be remarkably conserved across multicellular species such as fruit flies, nematodes, and mammals. The identified autophagy genes so far encode more than 20 autophagy core proteins, which have been functionally classified into five groups involved in the following autophagic processes: (1) the protein serine/threonine kinase complexes Atg1, Atg13, and Atg17 that act in the autophagy initiation phase; (2) the lipid kinase signaling complexes Atg6, Atg14, Atg34, and Vps15 that mediate phagophore formation; (3) two ubiquitin-like ligation systems (Atg8 and Atg12 systems) that promote autophagosome maturation; (4) Atg2, Atg9, and Atg18 that mediate the detachment and recycling of Atg proteins from autophagosomes after autophagosome maturation; and (5) the vacuolar integral membrane protein Atg22 that promotes the efflux of amino acids from degraded autophagosomes (Levine and Kroemer, 2008). The two ubiquitin-like conjugates mentioned above mediate the expansion and maturation of autophagosomes. The first is the Atg12–Atg5 conjugate, which is sequentially catalyzed by the E1-like enzyme Atg7 and the E2-like enzyme Atg10 and functions as a complex with Atg16L1. The second is phosphatidylethanolamine (PE)-conjugated Atg8 (microtubule-associated protein LC3 in mammals), which is activated by Atg7 and Atg3 (E2-like) enzymes (Levine et al., 2011). PE-bound LC3 (LC3-II) functions to bind lipid membranes and mediates the fusion and aggregation of lipid membranes. LC3-II is located on the membranes of autophagosomes and autolysosomes and is used as a universal marker of autophagy (Deretic and Levine, 2009).

Based on the selectivity of their cargo, autophagy can be divided into non-selective and selective autophagy. Under starvation or stress conditions, non-selective autophagy randomly engulfs and digests substances in the cytoplasm for rapid recycling to supplement inadequate nutrient uptake from the environment. Selective autophagy can specifically degrade protein aggregates, damaged organelles, excess peroxisomes, and invading pathogens to maintain intracellular homeostasis in non-starved cells. The selectivity of autophagy is achieved primarily through selective receptors on the surface of the substrate being degraded. LC3 on the surface of the autophagosome specifically binds to the selective receptors, thereby selectively isolating and enclosing the degraded substrate (Green and Levine, 2014).

## Mitophagy and its Molecular Mechanism

**FIGURE 1:**
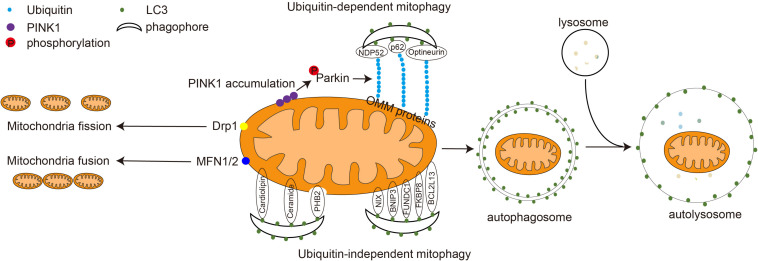
Mitochondrial dynamics and the molecular mechanism of mitophagy. In the physiological state, mitochondria fuse to form a tubular structure via the action of MFN1 and MFN2. In the pathological state, mitochondria are damaged and split into smaller granular mitochondria via the action of Drp1. The damaged mitochondria are degraded through either ubiquitin-dependent or ubiquitin-independent mitophagy. During ubiquitin-dependent mitophagy, PINK1 accumulates on the mitochondrial outer membrane, recruiting and activating the E3 ligase Parkin. Parkin ubiquitinates mitochondrial outer membrane proteins, including MFN1 and MFN2. The mitophagy adapter proteins p62, NDP52, and optineurin connect LC3 to ubiquitinated proteins on the OMM and guide mitochondria into the phagophore. In ubiquitin-independent mitophagy, mitophagy OMM receptors such as NIX, BNIP3, FUNDC1, BCL2L13, FKBP8, and IMM receptor PHB2, as well as mitochondrial lipids, including cardiolipin and ceramide, interact with LC3 directly. The phagophore gradually expands to form a double-layered autophagosome. Autophagosomes and lysosome fuse to form a single-layer-membrane-wrapped autolysosome to degrade and recycle the damaged mitochondrial substances.

Mitophagy is a form of selective autophagy in which dysfunctional mitochondria are specifically recognized and degraded, which is essential for mitochondrial quality control. In the initial phase of mitophagy, the mitochondria are directed to connect with a phagophore. The phagophore connected with the mitochondria expands into a double-layer autophagosome. Then the autophagosome fuses with the lysosome to form an autolysosome. Eventually, the mitochondria are degraded by the autolysosome (Figure 1). Recent studies have shown that mitophagy is involved in several processes such as development, cell death, and viral infection and that mitophagy malfunction is associated with major diseases such as neurodegenerative diseases, innate immune-related diseases, and cancer (Okamoto et al., 2009; Matthew et al., 2021).

The selectivity of mitophagy needs to be mediated by autophagy receptor/adaptor proteins. Although there is no clear difference between adapter and receptor, it is known that the receptor usually resides on the mitochondrial membrane, whereas the adapter is usually cytoplasmic and needs to be repositioned to the mitochondria during mitophagy. A variety of mammalian mitophagy receptors and adaptors have been reported, including OPTN, NDP52, FUNDC1, BNIP3, PHB2, NIX, p62, cardiolipin, and Bcl2L13 (Montava-Garriga and Ganley, 2020; Xie et al., 2020). These receptors contain the LC3 interaction region (LIR), a tetrapeptide sequence [W/F/Y]xx[L/I/V] motif that interacts with LC3. They can bind to LC3 and direct mitochondrial degradation via autophagy. Based on the adaptor/receptor involved and the requirement of ubiquitination, mitophagy can be broadly classified into two groups: ubiquitin-dependent and ubiquitin-independent mitochondrial autophagy (Figure 1; Stotland and Gottlieb, 2015).

### Ubiquitin-Dependent Mitophagy

Ubiquitin-dependent mitophagy is a crucial mechanism of mitochondrial quality control, and defects in this pathway are implicated in the pathogenesis of Parkinson’s disease. PTEN-induced putative kinase 1 (PINK1) is a serine/threonine kinase located in the inner mitochondrial membrane. In normal mitochondria, PINK1 entering the inner mitochondrial membrane is recognized and cleaved by the mitochondrial protease PARL and is degraded by the proteasome. In damaged mitochondria, PINK1 is stabilized on the OMM and recruits Parkin to promote mitophagy. Parkin is an E3 ubiquitin ligase located in the cytoplasm that catalyzes the covalent attachment of substrates to ubiquitin (Xie et al., 2020). Upon recruitment to damaged mitochondria, Parkin ubiquitinates some OMM proteins such as MFN1 and MFN2 (Figure 1). Adaptor proteins such as p62, NDP52, and optineurin recognize the polyubiquitinated protein on the OMM and tether mitochondria and the phagophore through the interaction between their LIR motif and LC3 (Matthew et al., 2021). Notably, some cells do not express PINK1 and Parkin, suggesting that either the presence of Parkin-like E3 ligase or the dependence of mitophagy on ubiquitination is cell and tissue specific (Williams et al., 2017).

### Ubiquitin-Independent Mitophagy

Ubiquitin-independent mitophagy is mainly mediated by mitophagy receptors, which are located on the mitochondria, contain a typical LIR motif, and can bind directly to LC3 (Figure 1; Pickles et al., 2018). NIX, also known as BNIP3L, contains LIR motifs that can interact with LC3, which is responsible for removing mitochondria during reticulocyte maturation. Mice deficient in NIX still have residual mitochondria in their mature erythrocytes (Novak et al., 2010). The homolog of NIX, BNIP3, also contains the LIR motif, which plays a role in hypoxia-induced mitophagy. In mouse cells lacking BNIP3, damaged mitochondria will continue to accumulate. Phosphorylation of BNIP3 Ser17 and Ser24 promotes its binding to LC3 and GATE16, thereby inducing mitophagy. Also, BNIP3 inhibits the fusion of damaged mitochondria, making it easier to eliminate them (Youle and Narendra, 2011). FUNDC1 is a tertiary transmembrane protein that is located on the OMM. It contains a typical LIR motif at its cytoplasm-facing N-terminus, which interacts with LC3 and mediates mitophagy under hypoxic conditions. Mammalian cells with FUNDC1 knockdown exhibit significantly impaired mitophagy in response to hypoxic stimulation (Liu et al., 2012). BCL2L13 is the mammalian homolog of the yeast mitophagy receptor Atg32. It contains two LIR motifs located at amino acid positions 147–150 and 273–276 and induces mitophagy through its interaction with LC3 (Otsu et al., 2015). Similarly, FKBP8 interacts with LC3 through the N-terminal LIR to induce mitophagy (Xie et al., 2020). In addition, ceramide is a lipid widely present on cell membranes, which can accumulate in the OMM under stress conditions and induce mitophagy by interacting with LC3 (Xie et al., 2020). When mitochondria are damaged, cardiolipin on the IMM flips to the OMM to interact with LC3 (Chu et al., 2013). The IMM protein PHB2 is an inner membrane mitophagy receptor, which is a component of the mitochondrial prohibitin complex. When mitochondria are damaged, the rupture of the outer membrane exposed PHB2 on the inner membrane to bind to LC3 and mediate mitophagy. During development, selective paternal mitochondrial elimination after fertilization promotes exclusive inheritance of maternal mtDNA. In *Caenorhabditis elegans*, paternal mtDNA is retained in the offspring when PHB2 is knocked down in males. Distinct paternal mitochondria are also retained in embryos from PHB2-knockdown males at the later stages of embryonic development (Wei et al., 2017).

## Triggering of Mitophagy During Viral Infection

**FIGURE 2:**
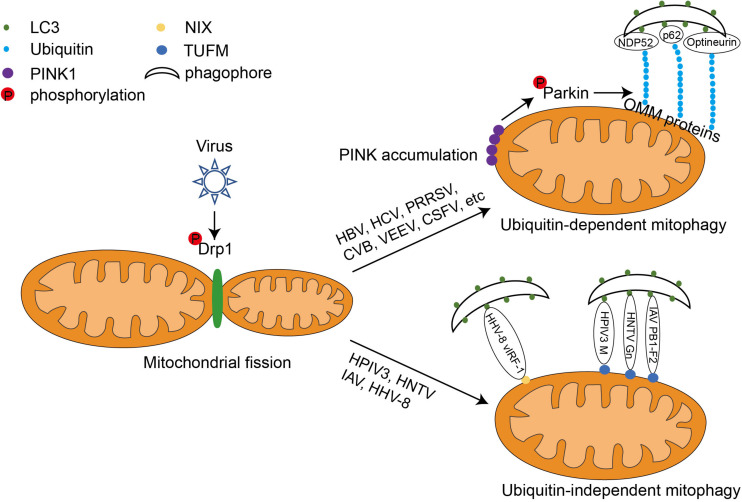
The mechanism of viral-induced mitophagy. Following viral infection, the GTPase Drp1 wraps around certain sites of mitochondria, forcing them to divide. This causes the mitochondria to separate from the tubular structure. Some viruses such as HBV and HCV utilize ubiquitin-dependent mitophagy. They upregulate the expression of PINK1 and Parkin, leading to the aggregation of PINK1 on the OMM, recruitment of the E3 ligase Parkin, and Parkin activation via phosphorylation. Activated Parkin can ubiquitinate OMM proteins such as MFN1 and MFN2. The poly-ubiquitinated OMM proteins serve as binding sites for both mitophagy adaptors (optineurin, p62, and NDP52) and LC3. The mitochondria can then be wrapped around by autophagosome and degraded by autophagy. Other viruses, such as IAV, HPIV3, and HTNV, can induce mitophagy via interactions with both LC3 and the mitochondrial elongation factor, TUFM. Interestingly, the HHV-8-encoded viral protein vIRF-1 directly induces NIX-mediated mitophagy in host cells.

Mitochondria are susceptible to the multifaceted effects of viral infection, either by direct attack of virus-encoded proteins or by regulation of infection-induced changes in cellular conditions. Therefore, maintaining a balance between mitochondrial fusion, fission, and the proper function of mitophagy is important for cells to cope with viral infections (Ren et al., 2020). Studies have shown that some viruses can manipulate various steps of the molecular machinery of mitophagy for their own benefit (Figure 2).

### Viral Proteins Induce Mitophagy by Increasing Mitochondrial Fission

Hepatitis B (HBV) and hepatitis C (HCV) viruses are infectious pathogens that pose a serious risk to human health and are major causative agents of liver cancer. Studies on HBV and HCV have shown that mitochondrial fission is required for mitophagy triggered by both viruses. HBV induces mitochondrial fission and promotes mitophagy by stimulating Drp1 phosphorylation at Ser616, suggesting the important role of Drp1 in Epstein–Barr virus (EBV)-induced mitophagy (Kim et al., 2013a; Khan et al., 2016; Burman et al., 2017). HCV also induces mitochondrial fission and mitophagy through a similar mechanism (Li et al., 2005; Kim et al., 2013b, 2014; Jassey et al., 2019). Ginsenoside G-Rg3 treatment reverses the HCV-induced mitophagy process by modifying the DRP1-mediated mitochondrial fission abnormalities caused by HCV infection (Kim et al., 2017). Furthermore, mitochondrial division and mitophagy induced by the PB1-F2 protein of influenza virus A were also inhibited when Drp1 was knocked down, suggesting that Drp1 plays an important role in initiating influenza-virus-induced mitophagy (Yoshizumi et al., 2014; Wang et al., 2021). Similarly, coxsackievirus B (CVB), Venezuelan equine encephalitis virus (VEEV), classical swine fever virus (CSFV), and porcine reproductive and respiratory syndrome virus (PRRSV) all trigger host cell mitophagy by promoting DRP1-mediated mitochondrial fragmentation (Li et al., 2016; Gou et al., 2017; Keck et al., 2017; Sin et al., 2017).

A recent study on polyomaviruses reported mitochondrial fragmentation as a conserved feature among the polyomaviruses BK polyomavirus (BKPyv), JCyv and SV40 (Manzetti et al., 2020). Another recent study on EBV infection showed that BHRF1, a BCL2 homolog encoded by EBV, also induces mitophagy by stimulating Drp1-mediated mitochondrial fission (Vilmen et al., 2020). Furthermore, in cells infected with HBV and CSFV, MFN2, which regulates mitochondrial fusion, is degraded by ubiquitination when mitochondrial fission is activated. The above studies suggest that the mitochondrial fission is a crucial upstream event for mitophagy after virus invasion. Modulation of mitochondrial fission, such as using drugs targeting Drp1, offers the potential for the discovery of new antiviral strategies.

### Viral Proteins Regulate Ubiquitin-Dependent Mitophagy

Ubiquitin-dependent mitophagy is dependent on the stabilization of PINK1 and its recruitment of Parkin to the OMM (Khaminets et al., 2016; Sato and Furuya, 2018). Many viruses have developed strategies to initiate mitophagy by activating the PINK1-Parkin duo. For example, HBV infection upregulates Parkin and PINK1 gene expression. In HBV-infected cells, Parkin-containing mitochondria have been observed to co-localize with the autophagosome marker GFP-LC3. The HBx protein of HBV is essential for HBV replication and propagation. It is mainly located in the cytoplasm and is also linked to mitochondria. Co-expressed exogenous Flag-HBx and mCherry-Parkin proteins physically interact with each other (Kim et al., 2013a). Similarly, HCV and its non-structural protein 5A induces the translocation of Parkin to mitochondria and upregulates the expression of Parkin and PINK1 in host cells (Kim et al., 2013b; Jassey et al., 2019). Furthermore, this is consistent with the elevated Parkin levels in the liver tissues of patients with chronic HCV (Cho et al., 2020). Conversely, the HCV core protein can interact with Parkin and inhibit its translocation to mitochondria, thus amplifying HCV-induced mitochondrial injury by suppressing mitophagy: this is consistent with the hepatocellular mitochondrial alterations in patients with chronic HCV (Hara et al., 2014; Jassey et al., 2019). Therefore, HCV has developed an effective pathogenic strategy by both inducing and antagonizing host mitophagy through different viral proteins. It has further been reported that EBV-encoded BHRF1 recruits Parkin and PINK1 to the mitochondria, and other viruses such as PRRSV, VEEV, and CSFV also induce host mitophagy in a similar manner (Figure 2; Li et al., 2016; Gou et al., 2017; Keck et al., 2017).

All the above examples suggest that viruses induce activation of ubiquitination-dependent mitophagy primarily by regulating the PINK1–Parkin pair. However, a recent study reported that the BKPyv agnoprotein regulates mitophagy in a Parkin-independent manner (Manzetti et al., 2020), which makes the relationship between ubiquitin-dependent mitophagy and viral invasion more complex.

### Viral Proteins Mediate Ubiquitin-Independent Mitophagy

In typical ubiquitin-independent mitophagy, the mitophagy receptors such as NIX, BNIP3, FUNDC1, and PHB2 use their classic LIR motif to bind LC3 on the phagophore directly to ensure selective degradation (Liu et al., 2014). Some viruses have evolved to use their encoded proteins as mitophagy adaptors to unite the mitophagy receptors and LC3. For example, human herpesvirus-8 (HHV-8) encodes vIRF-1, which is reported to bind directly to NIX to activate mitophagy (Vo et al., 2019). Other viral proteins, such as the matrix protein of human parainfluenza virus 3 (HPIV3), the PB1-F2 protein of influenza A virus (IAV), and the Gn protein of hantavirus (HTNV), all contain their own LIR motifs that act as junctions to connect TUFM and LC3 on the mitochondrial surface and direct mitochondria to the phagophore (Figure 2). Interestingly, TUFM is a protein with dual cytoplasmic and mitochondrial localization. It can be phosphorylated by PINK1 and recruited to the OMM; however, this process and the subsequent binding to viral junction proteins are not dependent on Parkin (Lin et al., 2020).

## Mitophagy and Host Antiviral Immunity

Both innate and adaptive immunity are involved in viral defense; simultaneously, viruses have evolved strategies to antagonize the host immune system. Innate immunity is the first line of defense against viral infection and is the basis of adaptive immune activation. In response to viral infection, innate immune cells secrete cytokines such as IFN to clear invaders and activate the adaptive immune system. Adaptive immunity, which is mediated by lymphocytes, produces immune memory and plays an irreplaceable role in the complete elimination of pathogens after reinfection (West et al., 2011; Hope and Bradley, 2021).

**FIGURE 3:**
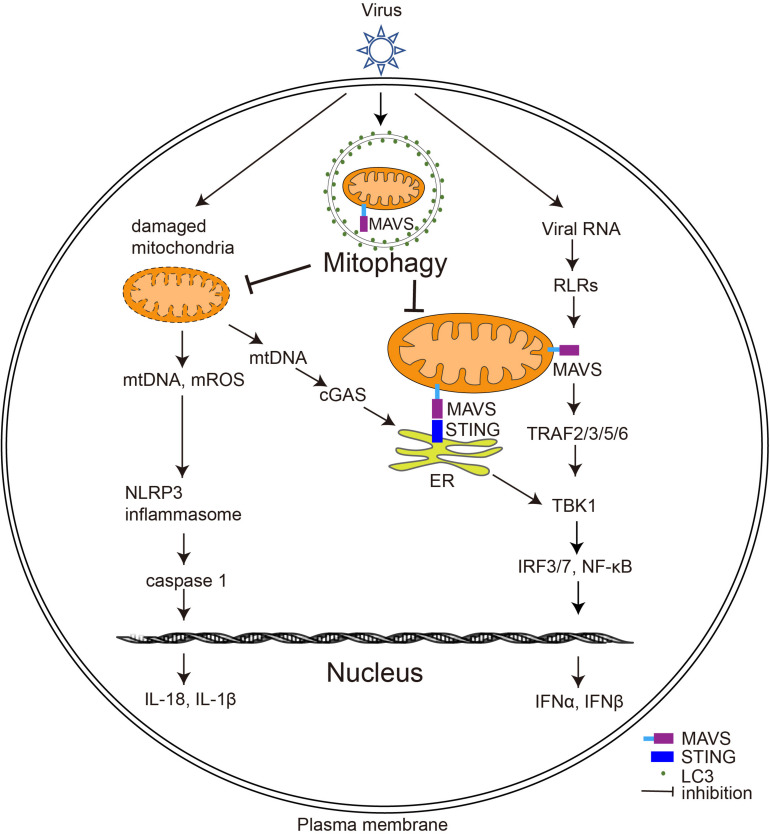
The effect of virus-induced mitophagy on the host immune system. Upon viral infection, RLRs (including MDA5 and RIG-I) on the host cell surface sense viral RNA and induce MAVS to form prion-like aggregates, which further trigger type I IFN production. Virus-induced mitophagy leads to the degradation of MAVS, thereby weakening the type I IFN response of the host. Furthermore, virus-induced mitophagy also removes damaged mitochondria to inhibit the activation of cGAS-STING signaling pathways and the inflammasome; ER, endoplasmic reticulum; mROS, mitochondrial ROS.

Given the critical role of mitochondria in immunity, viruses must manipulate the process of mitophagy at multiple levels to influence host antiviral immunity, including response to IFN, activation of the inflammasome, and activation of adaptive immunity. The following sections outline the functions of mitophagy and key components of mitophagy in the regulation of antiviral immune responses (Figure 3).

### Virus-Induced Mitophagy Dampens the Type I IFN Response

Interferons are proteins produced by a variety of cells in response to viruses or other IFN inducers. The production of type I IFNs (IFNs α and β) is a central element of the innate immune response to viruses and is crucial for host cell survival during viral infection (Perry et al., 2005). In response to viral infection, RIG-I-like receptors (RLRs), including MDA5 and RIG-I, sense viral RNA and induce MAVS to form prion-like aggregates and trigger downstream type I IFN production (Fan et al., 2020). MAVS-knockout mice are susceptible to infection by RNA viruses because their ability to produce type I IFNs is impaired (Sun et al., 2006).

Several viruses have developed strategies to suppress host immune responses by manipulating mitophagy to clear MAVS (Figure 3). HPIV3 utilizes its matrix proteins to induce mitophagy and facilitate MAVS degradation, thereby inhibiting type I IFN production (Ding et al., 2017). Similarly, measles virus (MeV), IAV, EBV, and HTNV dampen host type I IFN response by enhancing mitophagy function and attenuating MAVS (Xia et al., 2014a; Ding et al., 2017; Wang K. et al., 2019; Vilmen et al., 2020; Wang et al., 2021). In contrast, some viruses, such as HBV, directly destroy MAVS without autophagy by regulating key proteins involved in mitophagy. Although HBV is a DNA virus, its pre-genomic RNA can be sensed by RIG-I and activates MAVS sufficiently to mediate the synthesis of IFN-λ. Interestingly, HBV infection does not induce type I IFN production; the reason for this is that HBV infection massively increases the expression of Parkin, which interacts with MAVS and recruits the linear ubiquitin synthesis complex LUBAC to ubiquitinate MAVS, disrupting MAVS function and inhibiting IRF3 activation (Khan et al., 2016). Future studies could explore whether other viruses also degrade MAVS through mitophagy to weaken the host immune response.

### Virus-Induced Mitophagy and STING

In response to DNA virus invasion, cyclic GMP-AMP synthase (cGAS) is sensed and activated by the viral genomic DNA to catalyze the synthesis of cGAMP. cGAMP then functions as a second messenger, binding to the endoplasmic reticulum (ER) protein STING, which activates TBK1-IRF3 and triggers type I IFN production (Ishikawa et al., 2009; Sumpter and Levine, 2010).

EBV, a DNA virus belonging to the herpesvirus family, is closely linked with the pathogenesis of Burkitt’s and non-Hodgkin’s lymphoma. Overexpression of BHRF1, an early EBV-expressed lytic protein, induces mitophagy in HeLa cells and also blocks type I IFN production triggered by active STING and MAVS, suggesting a correlation between STING-and EBV-induced mitophagy; however, the mechanisms involved remain unclear (Vilmen et al., 2020). Human papillomavirus (HPV) is a small DNA virus that causes skin or mucous hyperplasia and encodes the E7 protein, which suppresses the immune response by inhibiting the cGAS-STING pathway. Based on the finding that the E7 protein activates Drp1 by targeting the retinoblastoma protein, promoting mitochondrial fission, and enhancing ceramide-mediated lethal mitophagy during chemotherapy, researchers have developed a mitophagy-inducing peptide that mimics the activation process of Drp1. Treatment of HPV-positive head and neck squamous cell carcinoma (HNSCC) cells with a combination of cisplatin and this new peptide synergistically increases cell mortality (Thomas et al., 2017), although the intrinsic link between E7-induced mitophagy and the inhibition of cGAS-STING during chemotherapy remains unclear.

### Mitophagy and Inflammation Upon Viral Invasion

In the innate immune system, pro-inflammatory factors and cytokines are involved in the elimination of viruses (Deretic and Levine, 2018). The inflammasomes consist of receptors and sensors of the innate immune system that regulate caspase-1 activation during viral infection (Lupfer et al., 2013; Guo et al., 2015). The NLRP3 inflammasome is one of the most intensely studied inflammasomes. Studies have shown that viruses, including IAV, vesicular stomatitis virus (VSV), and encephalomyocarditis virus, sequentially activate NLRP3 inflammasome and caspase 1, leading to the cleavage of inflammatory cytokine precursors and the maturation and secretion of IL-1β and IL-18 (Ichinohe et al., 2013).

Mice with knockout of Parkin, an important component of mitophagy, exhibit enhanced activation of NLRP3 inflammasome by mROS, elevated innate antiviral inflammation, and increased VSV clearance. However, NLRP3 deletion reverses the enhanced antiviral responsiveness in Parkin-knockout mice. Consistent with this, Parkin expression was found to be reduced in peripheral blood mononucleosis cells from virally infected patients. These results suggest that Parkin and its regulated mitophagy play an important role in antiviral immunity by controlling the mtROS-NLRP3-mediated inflammatory response (Li et al., 2019).

Receptor-interacting serine/threonine-protein kinase 2 (RIPK2) is a key molecule that mediates inflammation and regulates mitophagy by phosphorylating ULK1 (Honjo et al., 2021). *Ripk2-*knockout cells exhibit defective mitophagy, increased superoxide production, and accumulation of damaged mitochondria, leading to the activation of the NLRP3 inflammasome and production of IL-18 (Lupfer et al., 2013). Therefore, it is not surprising that *Ripk2*-knockout mice are susceptible to IAV infection (Lupfer et al., 2013). Consistent with the above studies, it has been found that an herbal compound called berberine suppresses IAV-triggered activation of the NLRP3 inflammasome of macrophages by inducing mitophagy to eliminate intracellular ROS. *In vivo* experiments further showed that the injection of berberine into IAV-infected mice blocks the induction of mitophagy and reduced inflammation (Liu et al., 2020), suggesting that interference with viral-induced mitophagy holds promise as a novel therapy to alleviate the symptoms of viral pneumonia.

The relationship between inflammation and mitophagy has also been studied in human immunodeficiency virus-associated neurocognitive disorders (HAND) that involve neuronal damage caused by HIV breaching the blood–cerebrospinal fluid barrier. The human immunodeficiency virus-1 (HIV-1) TAT protein is a transcription factor that is essential for the transactivation of HIV-1 transcription. Thangaraj and colleagues found that the TAT protein induces and leads to mitophagy volume aggregation and mitochondrial damage, which activates microglia and promotes the release of IL-1β, IL-18, and other inflammatory factors (Thangaraj et al., 2018). Thus, the modulation of mitophagy is expected to serve as a novel strategy for the treatment of HAND. The treatment of microglia with GU-rich single-stranded ssRNA40 derived from the HIV long repeat region has been shown to impede mitophagy, leading to the activation of the NLRP3 inflammasome and release of inflammatory cytokines, activating caspase 1 and causing to microglial pyroptosis (Rawat et al., 2019). Further in-depth studies in this direction are expected to elucidate the causes of cognitive disorders and chronic inflammation in patients with persistent HIV infections.

### Mitophagy and Antiviral Activity of the Adaptive Immune System

After a viral invasion, the adaptive immune system is capable of altering and adapting the immune response in response to reinfection with the same virus. Natural killer cells are innate lymphocytes with adaptive immune characteristics. They develop through three phases, namely, clonal expansion, contraction, and generation of long-lived “memory” cells. Chen’s group found that proliferating NK cells have increased damaged mitochondria after MCMV infection, but NIX-mediated mitophagy is induced during the contraction phase to maintain the survival of cells from the contraction phase to the memory generation phase (O’Sullivan et al., 2015). Their subsequent study has also found that Nix-mediated mitophagy promotes the survival of viral-specific CD8-positive memory T cells by regulating the ratio of long- and short-chain fatty acid oxidation. NIX knockout results in mitophagy defect and accumulation of mitochondrial peroxide and HIF1α, increased oxidation of short-chain fatty acids, and reduced ATP synthesis. This leads to a reduction of CD8-positive memory T cells and diminished immunity to viral reinfection (Gupta et al., 2019).

## Concluding Remarks

The focus on mitophagy as a type of cargo-specific autophagy has attracted significant attention in recent years, and its role in antiviral immunity is beginning to be revealed. Recent studies have shown that virus-induced mitophagy disrupts the signaling function of mitochondria in regulating the inflammatory response and immune response, leading to the establishment of persistent viral infection.

Although several efforts have been made in the last few years to understand the role of mitophagy in antiviral immunity, some questions remain to be clarified. For example, there is a need to investigate in greater depth the regulation of MAVS during viral invasion, as well as the strategies deployed by other viral proteins and viruses to induce and modulate mitophagy. Given that many viral proteins that induce mitophagy are essential for the replication of the virus itself, they have the potential to serve as major targets for antiviral therapy. In addition, the role of mitophagy in the survival of other natural lymphocytes following viral infection needs to be explored. In conclusion, further elucidation of the role of mitophagy in host antiviral immunity will deepen our understanding of the pathological process and provide new therapeutic strategies against viral invasion and associated diseases.

## Author Contributions

JL and HW were responsible for the conception and design of this study. HW, YZ, and JH conducted the literature search and summarization, and drafted the manuscript. JL revised the manuscript. All authors have read and approved the manuscript.

## Conflict of Interest

The authors declare that the research was conducted in the absence of any commercial or financial relationships that could be construed as a potential conflict of interest.

## Publisher’s Note

All claims expressed in this article are solely those of the authors and do not necessarily represent those of their affiliated organizations, or those of the publisher, the editors and the reviewers. Any product that may be evaluated in this article, or claim that may be made by its manufacturer, is not guaranteed or endorsed by the publisher.
